# A Bioequivalence Test by the Direct Comparison of Concentration-versus-Time Curves Using Local Polynomial Smoothers

**DOI:** 10.1155/2016/4680642

**Published:** 2016-12-05

**Authors:** Suyan Tian, Howard H. Chang, Dana Orange, Jingkai Gu, Mayte Suárez-Fariñas

**Affiliations:** ^1^Division of Clinical Research, First Hospital of Jilin University, 71 Xinmin Street, Changchun, Jilin 130021, China; ^2^Center for Clinical and Translational Science, The Rockefeller University, 1230 York Avenue, New York, NY 10065, USA; ^3^School of Mathematics, Jilin University, 2699 Qianjin Street, Changchun, Jilin 130012, China; ^4^Department of Biostatistics and Bioinformatics, Rollins School of Public Health, Emory University, 1518 Clifton Road NE, Atlanta, GA 30322, USA; ^5^Laboratory of Molecular Neurooncology, The Rockefeller University, 1230 York Avenue, New York, NY 10065, USA; ^6^Division of Rheumatology, Hospital for Special Surgery, 535 East 70th Street, New York, NY 10021, USA; ^7^Clinical Pharmacology Center, Research Institute of Translational Medicine, First Hospital of Jilin University, Dongminzhu Street, Changchun 130021, China; ^8^College of Life Science, Jilin University, 2699 Qianjin Street, Changchun 130012, China; ^9^Center for Biostatistics, Department of Population, Health Science and Policy, Icahn School of Medicine at Mount Sinai, New York, NY 10029, USA; ^10^Department of Genetics and Genomics Science, Icahn School of Medicine at Mount Sinai, New York, NY 10029, USA

## Abstract

In order to test if two chemically or pharmaceutically equivalent products have the same efficacy and/or toxicity, a bioequivalence (BE) study is conducted. The 80%/125% rule is the most commonly used criteria for BE and states that BE cannot be claimed unless the 90% CIs for the ratio of selected pharmacokinetics (PK) parameters of the tested to the reference drug are within 0.8 to 1.25. Considering that estimates of these PK parameters are derived from the concentration-versus-time curves, a direct comparison between these curves motivates an alternative and more flexible approach to test BE. Here, we propose to frame the BE test in terms of an equivalence of concentration-versus-time curves which are constructed using local polynomial smoother (LPS). A metric is presented to quantify the distance between the curves and its 90% CIs are calculated via bootstrapping. Then, we applied the proposed procedures to data from an animal study and found that BE between a generic drug and its brand name cannot be concluded, which was consistent with the results by applying the 80%/125% rule. However, the proposed procedure has the advantage of testing only on a single metric, instead of all PK parameters.

## 1. Introduction

In order to test if two chemically or pharmaceutically equivalent products, for example, a generic drug and its brand name, have the same efficacy and/or toxicity, a bioequivalence (BE) study is usually conducted [1–3]. The objective of a BE trial is to determine whether the test (*T*) and the reference (*R*) formulation of a pharmaceutical product are “equivalent” with respect to blood concentration × time profiles. In contrast to a difference test, the null hypothesis in an equivalence test states that two agents differ in terms of the endpoint under consideration by at least the minimum tolerable amount, called the equivalence margin Δ, whereas the alternative hypothesis states that such difference is less than the equivalence margin Δ:

(1) H0:μ≥Δ,versus  H1:μ<Δ.

In a BE test, some parameters derived from the concentration-versus-time curves are evaluated. Those parameters traditionally include the area under plasma concentration-versus-time curves (AUC_0_^*t*^), peak plasma concentrations (*C*_max⁡_), and its corresponding time (*T*_max⁡_). Among them, AUC is the most accepted measure of absorption rate. *C*_max⁡_ is also of importance because, for some drugs, a certain level of concentration needs to be reached to guarantee the desired therapeutic effect. *T*_max⁡_ is a relevant measure for drugs such as antibiotics that must reach the peak concentration quickly, but it may not be an appropriate measure for drugs requiring multiple dosage before a therapeutic effect is observed. Since none of these three parameters are universally superior to the others, a BE test usually considers them together. For example, under FDA regulations, BE can be claimed only when the 90% confidence intervals (CIs) for the ratio of *C*_max⁡_, AUC_(0−*t*)_, and AUC_(0−*∞*)_ of the tested (e.g., generic) to the reference drug (e.g., brand name) are within 80% to 125% [4]. This is referred to as 80%/125% rule, corresponding to a ±0.223 rule on the logarithmic scale (of the *T*/*R* ratio). Hence, the value of Δ is usually set to 0.223. Of note, the 90% CIs of the BE-endpoint represent a 0.05 significance level on the equivalence test since the hypothesis-testing problem in an equivalence test is divided into two one-sided hypothesis tests [2, 5, 6]: one where the null hypothesis states that the difference between two agents is less than −Δ whereas the other one assumes such difference is larger than Δ:

(2) H01:μ<−Δ,versus  Ha1:μ≥−Δ,H02:μ>Δ,versus  Ha2:μ≤Δ.

Although the BE parameters can be easily obtained from either one of the concentration-versus-time curves using a suitable pharmacokinetic (PK) model such as a one-compartment model or a nonparametric method [7], there are many parameters to be tested, which inflates the type I error rate, requiring the adjustment for multiple comparisons. Because these parameters may be highly correlated, such an adjustment is challenging.

On the other hand, some researchers had pointed out that the requirement of all confidence intervals falling within the equivalence bounds might lead to a conservative result, depending on the correlations among the PK parameters and the study power [8, 9]. To address this, simultaneous testing of all PK parameters had been explored and developed; see, for example, the semiparametric Bayesian approach proposed by Ghosh and Gönen [10]. However, such multivariate methods are less popular than the univariate approach of testing PK parameters, partially because of the modeling complexity associated with multivariate methods as compared with their univariate counterparts.

More importantly, when the confidence intervals of *C*_max⁡_, *T*_max⁡_, and AUC between two drugs all fall within the equivalence boundary (such that BE is concluded), it does not imply that the drugs are clinically equivalent since the overall shapes of the concentration-versus-time curves may in fact differ [11]. A falsely determined BE between two drugs when they are not clinically equivalent may be very harmful to the public. To alleviate these limitations and drawbacks, we propose to make a direct comparison of the concentration-versus-time curves that accounts for the differences between the profile shapes for BE testing. The main objective of this paper is to present a strategy to test BE with the aid of local polynomial smoother (LPS), which is used to construct concentration-versus-time curves. Then, a summary statistic, whose standard error is estimated by bootstrapping, is defined. This allows the calculation of CIs upon which decisions over equivalence between such two curves are made.

LPS is a flexible nonparametric regression method to model curves or surfaces. Specifically, the fitted regression function at a covariate value *x* is based only on observations within a prespecified neighborhood of *x*. LPS can be traced back to the late 19th century in actuarial sciences where it was used to estimate the gradation of mortality rates and in time series modeling [12]. In contrast, another popular nonparametric regression method, locally weighted scatterplot smoothing (LOWESS), obtained smooth fitted values by a weighted linear least squares regression over the prespecified spans. In this study, however, we do not emphasize their differences and instead treat them as synonymous.

With advances in computing, the potential applications of LPS continue to expand. For example, it has been used for dye normalization of two-color microarray experiments [13]. Recently, Anders and Huber [14] used LPS in R's DESeq package to estimate the mean-variance relationship in a negative binomial model, a technique now commonly used to model RNA-Seq data. The popularity of LPS in statistical applications may be due to its several advantages including satisfactory boundary behavior and straightforward interpretability of the nonlinear relationships [15, 16].

## 2. Methods and Materials

### 2.1. Experimental Data

The study included 24 beagle dogs weighting 9–11 kg, and those dogs were randomly assigned to receive either single or multiple (every four weeks for 3 times) administrations of either 1.4 mg/kg Sandostatin or a generic drug developed by GenSci. There were 6 dogs including 3 females and 3 males per treatment-dosage group. Blood samples for PK were collected into tubes containing heparin sodium and centrifuged at room temperature with 3500 rpm for 20 min to obtain plasma samples. This animal study was conducted in accordance with the rules and regulations of Chinese Pharmacopoeia, 2000 Edition/Version 2 and approved by the Committee on the Ethics of Animal Experiments of Jilin University. The plasma concentrations were determined using an Agilent 1100 liquid chromatography-tandem mass spectrometry. In the single-dose regimen, blood samples were collected at the initial time corresponding to hour 0 and at the hours of 0.25, 0.5, 1, 1.5, 2, 4, 8, 24, 48, 96, 144, 192, 240, 336, 432, 528, 624, 720, 816, 912, 1008, 1104, and 1200.

Based on [7], PK parameters in this study were calculated as follows. For each dog, the maximum plasma concentration (*C*_max⁡_) and its corresponding time (*T*_max⁡_) were determined by visual inspection of the profiles. The apparent terminal elimination rate constant (*λ*) was calculated by linear regression of the natural logarithms of the terminal plasma concentrations. The terminal half-life (*t*_1/2_) was derived as 0.693/*λ*. The area under the curve (AUC) to the last measured point (AUC_0_^*t*^) was calculated using the trapezoidal rule. The AUC for the plasma concentration-versus-time function from 0 hours to infinity (AUC_0−*∞*_) was calculated as the sum of AUC_0_^*t*^ and *C*_*t*_/*λ*, while *C*_*t*_ was the last quantifiable concentration.

### 2.2. The LPS Model

Let (*X*_1_, *Y*_1_),…, (*X*_*n*_, *Y*_*n*_) be covariate-response pairs for *n* observations. To simplify the notation, suppose there is only one covariate. Then, the response *Y*_*i*_ is related to the covariate *X*_*i*_ through the following model:

(3) $$\begin{array}{c} Y_{i}=g\left(\;X_{i}\;\right)+\varepsilon_{i},\;i=1,\ldots ,n, \end{array}$$

where the error term *ε*_*i*_ is assumed to be independently distributed with zero mean and variance equal to *σ* ^2^. The goal of a local polynomial smoother is to estimate the smooth function *g*(*x*) = *E*(*Y*∣*X* = *x*) using an approximation provided by a local polynomial of low order in the neighborhood of the point *x*. Usually, the model includes only first- and second-degree polynomials, that is, either locally linear or locally quadratic. This is because any function can be well approximated in a small neighborhood by low-order polynomials and the inclusion of higher order terms often does not improve the model fit dramatically and even may lead to overfitting instead. Specifically, for *x* − *h* ≤ *x*_*i*_ ≤ *x* + *h*,

(4) $$\begin{array}{c} g\left(\;x_{i}\;\right)\approx \overset{p}{\underset{j=0}{\sum }}\beta_{j}{\left(\;x_{i}-x\;\right)}^{j}. \end{array}$$

The abovementioned approximation is fitted by local weighted least squares, where the estimated coefficients are those that minimize the following function:

(5) $$\begin{array}{c} \overset{n}{\underset{i=1}{\sum }}W\left(\;\frac{\left(x_{i}-x\right)}{h}\;\right){\left(\;y_{i}-\sum_{j=0}^{p}\beta_{j}{\left(\;x_{i}-x\;\right)}^{j}\;\right)}^{2}, \end{array}$$

where the weighted function *W* gives greater weight to *x*_*i*_ in the vicinity of *x* and is usually specified as a symmetric bounded function such as a normal kernel function.

There are two commonly used approaches for the selection of bandwidth *h* in the equation above [12]. One choice is simply to make the bandwidth a constant, which is adequate if the regression function behaves smoothly. The other one is to allow the bandwidth to change as a function of *x*. In our applications, we chose the latter by using a nearest-neighbor bandwidth selection method [17]. Briefly, in the nearest-neighbor bandwidth selection method, a new parameter *α* was included, which was multiplied by the sample size *n* and rounded up to an integer *k*. Then, the bandwidth *h*(*x*) was defined as the distance from *x* to the *k*th closest *x*_*i*_. In this study, measurements were taken at nonuniformly spaced time points, with more frequent measurements at the beginning, where a larger variation of the PK dynamic was expected among subjects. Under this condition, the nearest-neighbor bandwidth selection approach will provide better fit than the constant bandwidth. The nearest-neighbor bandwidth parameter *α* was determined by a leave-one-out (LOO) cross-validation due to the small sample size. Specifically, for a grid of possible *α* values, cross-validated mean square error (MSE) was calculated, and the *α* corresponding to the minimum MSE was selected.

### 2.3. Statistical Language

The statistical analysis was carried out in the R language version 2.15 (http://www.r-project.org/). The R-code is available upon request.

## 3. Results and Discussion

### 3.1. Testing the Equivalence of Two Curves by Combining LPS and Bootstrapping: The Proposed Procedure

Here, we propose to evaluate bioequivalence between drugs by directly comparing the plasma concentration curves PC(*t*). Suppose we have two treatments where *n*_1_ subjects were randomly assigned to receive the test drug *T* and *n*_2_ to the reference drug *R*. We proposed to reframe the hypothesis represented in (1) as

(6) H0:dgTt,gRt>Δ,

(7) versus  H1:dgTt,gRt≤Δ,

with *d* representing the difference between the PK curves for *T* and *R* drugs. For the study subjects, the plasma concentrations of these drugs were measured for those at a grid of time points. Using LPS (details in Section 2), concentration-versus-time curves for both treatments can be estimated as *g*_*R*_(*x*) and *g*_*T*_(*x*). To evaluate if the differences between two curves are within the tolerable range, we define the following estimator of the average difference between the two curves at a grid of prespecified time points, for example, the time points where the concentrations were taken in this study:

(8) ln⁡r^=∑k=1Kln⁡y^Ttk/y^RtkK,

where ${\hat{y}}_{Tt_{k}}$ and ${\hat{y}}_{Rt_{k}}$ are the fitted values of plasma concentration using LPS at time *t*_*k*_ for the test drug *T* and the reference drug *R*, respectively. Here, *k* = 1,2,…, *K*, and *K* is the number of time points at which the difference between two curves is evaluated. Note that ln⁡(*r*) is a measure of the difference between the curves and when the two curves are identical, and they should be identical at all time points and thus the value of ln⁡(*r*) should be 0.

The standard error for $\ln\left(\hat{r}\right)$ can be estimated using bootstrapping [18]. For each bootstrapped replicate, LPS curve was fitted for *T* and *R* and the difference between them is calculated using (8). The 90% CIs of *r* can be obtained as

(9) $$\begin{array}{c} \left(\;e^{\ln\left(\hat{r}\right)-1.645\times se\left(\ln\left(\hat{r}\right)\right)},e^{\ln\left(\hat{r}\right)+1.645\times se\left(\ln\left(\hat{r}\right)\right)}\;\right). \end{array}$$

If the 90% CIs is within 0.8~1.25, then the equivalence between two curves is claimed.

### 3.2. Case Study

Octreotide is an octapeptide that mimics natural somatostatin pharmacologically even though it is a more potent inhibitor of growth hormone, glucagon, and insulin than the natural hormone. Brand name Sandostatin (Novartis Pharmaceuticals) of octreotide had been approved for the treatment of several diseases, such as acromegaly and gigantism. To evaluate the BE between a generic octreotide developed by GenSci Pharmaceuticals (Changchun, China) and Brand name Sandostatin, a pilot study using 24 beagle dogs was conducted. The generic drug is referred to as GenSci hereafter. Using this study as an example, we showcase how to incorporate LPS seamlessly with other statistical methods to test BE. Once concentration-versus-time curves are obtained via LPS, testing for BE will be akin to testing if the two LPS-derived curves are the same.

**Figure 1:**
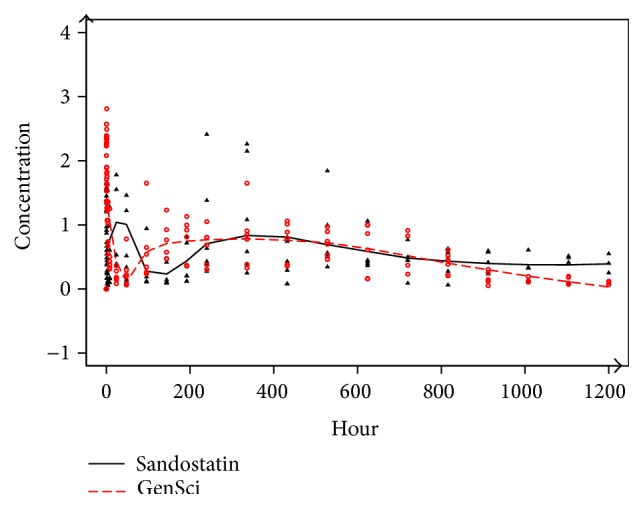
The fitted local polynomial smoother (LPS) curves for both GenSci and Sandostatin. Black triangle: the observed concentrations for dogs using Sandostatin; red circle: the observed concentrations for dogs using GenSci.

Firstly, we conducted an equivalence test using the procedure described in Methods. The fitted plasma concentration PC(*t*) curves are shown in Figure 1. Interestingly, the curves estimated by LPS suggested some PK differences between the two drugs. Specifically, we observed a surge in plasma concentration and then a steep drop in the GenSci group. In addition, the GenSci group entered the plateau stage faster and remained in this stage longer than the Sandostatin group, dropping down at a sharper scope. Based on these observations, we suspected that GenSci might have a better PK behavior than Sandostatin.

The 90% CIs for the proposed metric of GenSci against Sandostatin were calculated as 119.02%~166.99%. With its upper bound above 125%, the equivalence between the two drugs cannot be established based on this method. As a comparison, we also estimated the 90% CI of the ratio of the traditionally considered PK parameters (i.e., (AUC_0_^*t*^), *C*_max⁡_, and *T*_max⁡_) based on the FDA guidelines [4]. The results summarized in Table 1 show that all 90% CIs of the ratio of (AUC_0_^*t*^), *C*_max⁡_, and *T*_max⁡_ of GenSci against Sandostatin were outside 80% to 125%, the FDA-sanctioned BE criteria. In summary, BE cannot be established using either the FDA-criteria or our proposed procedure. Nevertheless, the proposed procedure has the advantage of testing only a single statistic and hence avoiding multiple hypotheses issues.

The sample size of 6 dogs per group raises major concerns in light of the failure to establish BE between GenSci and Sandostatin. To explore the impact of the sample size in this pilot study, we combined the data from this single-dose cohort with additional data from a multiple-dose cohort, but including only data collected in the first 720 hours where no boosts were given and the experimental methods∖conditions were exactly the same as in the single-dose cohorts. Interested readers may see Section 2 for more information on how the experiments were conducted. Analysis on the combined cohorts including 12 dogs per group led to similar results. Given that this application represents a pilot study, as preliminary work for larger studies, no confirmative conclusions were drawn here. By presenting this study, our intent is to illustrate the LPS-based BE test as an alternative to the classical univariate tests on PK parameters.

### 3.3. Simulation Study

We conducted a simulation study to characterize our BE-testing procedure. Here, we used the PK model presented in [11] where the concentration function is represented by

(10) $$\begin{array}{c} c\left(\;t\;\right)\propto \frac{K_{a}}{K_{a}-K_{e}}\left(\;e^{-k_{e}t}-e^{-k_{a}t}\;\right), \end{array}$$

where *k*_*a*_ and *k*_*e*_ are the absorption and the elimination rate constants. The concentration-versus-time curves for the reference drug and test drug were simulated as

(11) log⁡YR=log⁡ct+ε,log⁡YT=log⁡ct+∇+ε,ε~MVN0p,Σp×p,

where the correlation between two measures, on the logarithm scale, from the same subject *k*  (*k* = 1,2,…, 2*n*) was specified as a positive constant; that is, corr⁡(*ε*_*ik*_, *ε*_*jk*_) = *ρ*  for  *i* ≠ *j*. The curves above differ only by a constant ∇ on the logarithm scale. In the second set of simulations, the two concentration curves for the test drug and the reference drug were set to have different absorption rates (i.e., *k*_*aT*_ and *k*_*aR*_) instead of ∇≠0.

By varying the values for *ρ*, ∇, and *k*_*aT*_/*k*_*aR*_ and the sample size, we simulated 100 different scenarios in total. The empirical coverage of the 90% CIs for each scenario was calculated via simulating 1000 bootstrap replicates. The simulation results are presented in Table 2, from which we found that a sample size of 24 per group is large enough to provide an adequate statistical power for the proposed testing procedure. Furthermore, the empirical coverage of the 90% CIs when two curves are equivalent is approximately 95% or above, suggesting the 80/125% rule is applicable for the proposed metric.

### 3.4. Our Extension to the Superiority Test between Curves

It is worth pointing out that by virtue of being coupled with a permutation test [19, 20] to calculate an overall *p* value, LPS can also be used to evaluate if two curves are different. In this case, the hypothesis tested is

(12) H0:dgAx,gBx=0,versus  H1:dgAx,gBx≠0,

with *d* representing the difference between the two curves. As shown on the simulation studies (in Supplementary Material available online at http://dx.doi.org/10.1155/2016/4680642), the permutation-based test provides a valid means for testing the difference of two curves, achieving the desired type I error rate. We applied this procedure to longitudinal data collected by Orange et al. [21] and showed that the results obtained were consistent with those obtained by using a generalized estimating equation model [22, 23]. Therefore, the proposed procedure to test the difference between curves using LPS can be employed in a wide range of applications in longitudinal data analysis.

## 4. Conclusions

In this study, we presented a framework which directly compares the concentration-versus-time curves constructed using LPS to test BE. This approach avoids multiple testing by evaluating the equivalence between curves with one single metric, instead of multiple PK parameters. Furthermore, since LPS can be viewed as an extension of linear weighted regression and can be easily implemented in any standard statistical software, our proposed procedure can be easily utilized in other applications.

van der Meersch et al. [24] have argued that the objectives and methodology of bioequivalence trials differ greatly from those of noninferiority and equivalence trials due to the decision criteria and study design. In our opinion, their claims are not persuasive. In terms of decision criteria, it is difficult to define an equivalence margin in a longitudinal sense because the measures such as plasma concentrations are collected over time. It might represent one major reason why the FDA and other regulatory agencies have defined BE by using confidence limits for a grid of PK parameters instead of a single hypothesis testing. Regarding the study design, van der Meersch et al. [24] deemed a crossover design to be suitable and appropriate. In practice, however, there are many BE studies using a parallel design that the FDA reports [4] explicitly listed the cases where a parallel design is considered more suitable than a crossover design. Given that a BE test is a longitudinal equivalence test by nature, we believe that the use of nonparametric smoothers such as LPS as a basis to construct the concentration-versus-time curves for direct comparisons warrants additional attention and research.

## Supplementary Material

The case study illustrates how to test the difference among curves with the aids of LPS.

## Figures and Tables

**Table 1:** The 90% CIs of PK parameters.

90% CI	Single dose	Combined data
	*N* = 6	*N* = 12
*C*_max⁡_	(72.23, 160.71)	(82.93, 160.88)
*T*_0.3 ng/nL_	(9.94, 382.63)	(9.51, 144.60)
AUC_0–1200_	(71.98, 176.08)	—
AUC_0–720_	(61.74, 169.85)	(91.44, 208.50)

**Table 2:** The exact coverage of the 90% CIs for ln⁡(*r*) (the proposed measure of the difference between two curves) using simulations.

	*n* = 12	*n* = 18	*n* = 24	*n* = 30	*n* = 50
∇ = 0, *k*_*aT*_/*k*_*aR*_ = 1 (two curves are identical)					
*ρ* = 0	100	100	100	100	100
*ρ* = 0.2	100	100	100	100	100
*ρ* = 0.5	99.9	100	100	100	100
*ρ* = 0.8	97.8	99.6	100	100	100

∇ = 0.223, *k*_*aT*_/*k*_*aR*_ = 1 (the difference is within the equivalence bound)					
*ρ* = 0	100	100	100	100	100
*ρ* = 0.2	100	100	100	100	100
*ρ* = 0.5	98.7	99.7	100	100	100
*ρ* = 0.8	95.5	97.2	99.1	99.7	100

∇ = 1, *k*_*aT*_/*k*_*aR*_ = 1 (the difference is beyond the equivalence bound)					
*ρ* = 0	0	0	0	0	0
*ρ* = 0.2	3.3	0.7	0.3	0	0
*ρ* = 0.5	11.6	6.6	3.7	3.2	0.7
*ρ* = 0.8	18	11.2	7.9	5.8	2.6

*k*_*aT*_/*k*_*aR*_ = 1.25, ∇ = 0 (the difference is within the equivalence bound)					
*ρ* = 0	100	100	100	100	100
*ρ* = 0.2	99.9	100	100	100	100
*ρ* = 0.5	97.7	99.1	99.7	100	100
*ρ* = 0.8	95	97.5	98.8	99.3	100

*k*_*aT*_/*k*_*aR*_ = 3, ∇ = 0 (the difference is beyond the equivalence bound)					
*ρ* = 0	0	0	0	0	0
*ρ* = 0.2	5	1.5	0.6	0.4	0
*ρ* = 0.5	13.2	8.2	5.4	3.8	0.7
*ρ* = 0.8	17.7	15.8	10.2	6.5	2.7

Note: *k*_*aT*_ represents the absorption rate for the generic drug and *k*_*aR*_ represents the absorption rate for the reference drug; *n* represents the sample size of each group; ∇ represents a constant difference between the concentrations of two drugs on the logarithm scale; *ρ* is the correlation coefficient between measures over time from the same subject; its influence on the estimation is expected to be bigger when its value is larger.
