# Supplementary material for: A Bioequivalence Test by the Direct Comparison of Concentration-versus-Time Curves Using Local Polynomial Smoothers
Source: Comput Math Methods Med. 2016 Dec 5;2016:4680642. doi: 10.1155/2016/4680642 (PMC5165228; doi:10.1155/2016/4680642)
Supplement: Supplementary file 1 — The case study illustrates how to test the difference among curves with the aids of LPS. [file 4680642.f1.doc]

**Supplementary file: The difference test between curves using local polynomial smoothers (LPS).**

## The difference test between two curves using LPS and permutation tests

The basic concept of a permutation test is to construct the distribution under null hypothesis by recalculating a test statistic such as T-value via many permutations of the original data [1, 2]. In this paper, we used a permutation test to calculate a p-value on which we evaluate if the null hypothesis of no difference between two curves should be rejected.

The concentration versus time curves for both treatments can be estimated as g_A_(t) and g_B_(t). Then, the distances between them are calculated as the squared Euclidean distance between g_A_(t_i_) and g_B_(t_i_) at a set of pre-determined time points t_1_…. t_K_ and those distances are accumulated, i.e,

where $\hat{y}_{At_{k}}$and $\hat{y}_{Bt_{k}}$are the fitted values of plasma concentration using LPS at time t_k_ for treatment A and B, respectively. The empirical distribution of metric D under the null hypothesis can be obtained by a permutation test, where the treatment assignments were permuted *M* times, thus the observations from the same sample were not perturbed. Then g_Am_(t) and g_Bm_(t) curves were estimated via LPS again, and the distance D_m_ (m=1,…M) was calculated for each permutation. A p-value, the probability of getting a test statistic as extreme as or more extreme than the observed test statistic D under the null hypothesis, can be calculated as,

.

## Simulations

To evaluate if the proposed difference test of two curves possesses the desired statistical properties for detecting difference between two curves, we conducted a simulation study under 4 different scenarios.

In the first scenario, the two curves were simulated as 2n independent identical distributed (iid) random variables Y_1_ and Y_2_ differing only by a constant, i.e.:

$$\begin{matrix} Y_{1}=f(x)+\varepsilon_{1},\varepsilon_{1}\sim N(0,1) \\ Y_{2}=f(x)+\Delta+\varepsilon_{2},\varepsilon_{2}\sim N(0,1) \\ f(x)=0.5x^{3}+0.4x+1,x=1,1.01,...,10 \\ \end{matrix}$$

In the second scenario, the setting for Y_1_ and Y_2_ are the same as above except that Δ is not a constant. Instead, the values of Δ changed linearly as a function of x. For example, Δ=1 represents the difference between Y_1_ and Y_2_ is -1 when x=min(x); such difference is 1 when x=max(x); the scope is 2Δ/(x_max_-x_min_)=2/9. In this case, the differences between two curves are the largest at the tails and these two curves intersect in the middle.

In the third and fourth scenarios, the underlying function f(x) and Δ were the same as the two previous simulations with a fixed value for Δ set to be 1.5 and 2.5, respectively. However, the correlation between two measures from the same subject was set as a positive constant, i.e., . With the third and fourth simulations, we explored the effect of the correlation between the observations from the same subject and its effects on the permutation-based p-value.

In all simulations, we used LPS to estimate the underlying curves and the permutation test (m=500) to calculate the corresponding p-value (See the Methods section for details). We found that when Δ is small, the resulting p-values were often greater than 0.05. When Δ is reasonable large, the p-values were able to indicate the statistically significant difference between two curves. The role of the sample size played here is consistent with the large-sample statistical theory.

By setting Δ=0, we also verified that the permutation-based p-value indeed achieved the desired type I error rate (e.g., for n=20 in each group, we repeated the simulation 100 times, among the resulting p-values, there are 6 of them less than 0.05). Thus, this procedure provides indeed a valid metric to detect the difference between two curves.

## Real world application

FK506 (Tacrolimus) is a potent inhibitor of calcineurin that leads to death of activated T cells and is used to treat autoimmunity and to prevent transplant rejection. Dendritic cells (DCs) are critical regulators of T cell mediated inflammation and tolerance. DCs loaded with FK506 (FKDC) are potent inhibitors of T cell responses. To test the effect of FKDC in an in-vivo model of autoimmunity, mice with arthritis were treated under 4 treatment arms (A: FKDC pulsed with relevant type II collagen, B: FKDC pulsed with irrelevant type I collagen, C: untreated DC pulsed with CII, D: no DC at all). The observed onset rates of moderately severe arthritis (arthritis severity score > 5) under these regimes were compared in 3 separate, sequential experiments. Briefly, in the first experiment there were 3-5 mice per group. This experiment was conducted at the end of 2010, and arthritis severity scores were measured on day 0, 3, 6, 10, 12, and, 17. In the second experiment, conducted in the period of March-June, 2011, there were 5 mice per group and arthritis measurements were taken at day 0, 10, 15, 18, 22, 25, 28, 32, 35, 39, 42, 44, 46, 49, 51, 67, 73, 81, and 87. The third experiment included about 8-12 mice per group and arthritis severity was measured at day 0, 3, 5, 7, 10, 13, 15, 18, 20, 22, 25, 27, 29, 33, 35, 47, 54, and 61.

Since time points of arthritis measurement varied among these 3 experiments, pooling the data from each experiment together was difficult. LPS provided an effective way to utilize all available data, while considering the non-uniformity of the time points. Specifically, after combining the data from three experiments into one, the incidence rates stratified by the treatment groups at each time point were calculated. Upon the calculated incidence rates, LPS curves were constructed and the distances among pairs of curves were calculated. The curves given by these smoothers could be regarded as the trajectory of incidence rates (how the incidence rates changed over time) for each treatment stratum (Supp. Figure 1).

The pair-wise comparisons between those treatments whose significance was assessed via permutation test (m=1000) indicate that the incidence of severe arthritis is significantly lower in mice treated with drug A than with the control regimes (p <0.001, <0.001, and 0.002 for B, C, and D, respectively). These results were in consistent to the results presented in [3].

Supplementary Figure 1. The fitted local polynomial smoother (LPS) curves using the data from the Orange’s study

**References**

1. Good PI: *Permutation, Parametric and Bootstrap Tests of Hypotheses*. 3rd edition. Springer; 2005.

2. Welch WJ: **Construction of permutation tests**. *J Am Stat Assoc* 1990, **85**:693–698.

3. Orange DE, Blachere NE, Fak J, Parveen S, Frank MO, Herre M, Tian S, Monette S, Darnell RB: **Dendritic cells loaded with FK506 kill T cells in an antigen-specific manner and prevent autoimmunity in vivo.** *Elife* 2013, **2**:e00105.
